# Echinochrome A Increases Mitochondrial Mass and Function by Modulating Mitochondrial Biogenesis Regulatory Genes

**DOI:** 10.3390/md12084602

**Published:** 2014-08-21

**Authors:** Seung Hun Jeong, Hyoung Kyu Kim, In-Sung Song, Su Jin Noh, Jubert Marquez, Kyung Soo Ko, Byoung Doo Rhee, Nari Kim, Natalia P. Mishchenko, Sergey A. Fedoreyev, Valentin A. Stonik, Jin Han

**Affiliations:** 1 Cardiovascular and Metabolic Disease Center (CMDC), National Research Laboratory for Mitochondrial Signaling, Inje University, Busan 614-735, Korea; E-Mails: shejeong96@gmail.com (S.H.J.); estrus74@gmail.com (H.K.K.); microvirus@hanmail.net (I.-S.S.); kskomd@paik.ac.kr (K.S.K.); bdrhee@hanmail.net (B.D.R.); narikim43@gmail.com (N.K.); 2 Department of Physiology, College of Medicine, Inje University, Busan 614-735, Korea; 3 Department of Health Sciences and Technology, Graduate School of Inje University, Busan 614-735, Korea; E-Mails: msns6336@gmail.com (S.J.N.); jcuevas.marquez@gmail.com (J.M.); 4 George B. Elyakov Pacific Institute of Bioorganic Chemistry, Far-Eastern Branch of the Russian Academy of Science, Prospect 100 let Vladivostoku, 159, Vladivostok 690022, Russia; E-Mails: mischenkonp@mail.ru (N.P.M.); fedoreev-s@mail.ru (S.A.F.); stonik@piboc.dvo.ru (V.A.S.)

**Keywords:** echinochrome A, mitochondrial biogenesis, oxygen consumption rate

## Abstract

Echinochrome A (Ech A) is a natural pigment from sea urchins that has been reported to have antioxidant properties and a cardio protective effect against ischemia reperfusion injury. In this study, we ascertained whether Ech A enhances the mitochondrial biogenesis and oxidative phosphorylation in rat cardio myoblast H9c2 cells. To study the effects of Ech A on mitochondrial biogenesis, we measured mitochondrial mass, level of oxidative phosphorylation, and mitochondrial biogenesis regulatory gene expression. Ech A treatment did not induce cytotoxicity. However, Ech A treatment enhanced oxygen consumption rate and mitochondrial ATP level. Likewise, Ech A treatment increased mitochondrial contents in H9c2 cells. Furthermore, Ech A treatment up-regulated biogenesis of regulatory transcription genes, including proliferator-activated receptor gamma co-activator (PGC)-1α, estrogen-related receptor (ERR)-α, peroxisome proliferator-activator receptor (PPAR)-γ, and nuclear respiratory factor (NRF)-1 and such mitochondrial transcription regulatory genes as mitochondrial transcriptional factor A (TFAM), mitochondrial transcription factor B2 (TFB2M), mitochondrial DNA direct polymerase (POLMRT), single strand binding protein (SSBP) and Tu translation elongation factor (TUFM). In conclusion, these data suggest that Ech A is a potentiated marine drug which enhances mitochondrial biogenesis.

## 1. Introduction

Sea urchins, which belong to marine biocenosis, produce a number of unique substances, such as quinonoid pigments named spinochromes [1,2]. Of these compounds, echinochrome A (Ech A) possesses the highest antioxidant activity and is the most common dark-red pigment of sea urchin shells, spines, and eggs [2,3]. Ech A can act through a number of antioxidant mechanisms, including the scavenging of active oxygen radicals [4], interaction with lipoperoxide radicals [5], chelation of metal ions [6], inhibition of lipid peroxidation [7], and regulation of the cell redox potential [8]. Its chemical structure, named 6-ethyl-2,3,5,7,8-pentahydroxy-1,4-naphthoquinone, was confirmed by X-ray analysis [9]. This natural compound is soluble in ethanol and other organic solvents and insoluble in water. Ech A is an active substance (P N002362/01) in some medicines called Histochrome^®^ in Russia, and was patented in other countries as an active substance in certain drugs [10,11,12].

Besides antioxidant properties, Ech A has a cardioprotective effect against reperfusion injury [13]. The Histochrome^®^ drug is produced as an isotonic solution of di- and trisodium salts of Ech A at a concentration of 0.2 mg/mL. Histochrome^®^ normalizes metabolic processes and eliminates inflammation in the retina, vascular membrane, and cornea of the eye. It improves trophic functions, reduces edema, and accelerates epithelialization, when administered into patients [14]. However, its protection and regulation mechanisms in mitochondria are unclear.

Mitochondria are essential and pivotal regulators of cellular bioenergetics [15]. Mitochondria function to maintain homeostasis in response to environmental stimuli, such as hormones, nutrients and oxygen tension [16,17,18,19]. Cardiomyocyte mitochondria respond to rapid, temporary changes in oxygen and Ca^2+^ levels during ischemic reperfusion [20,21]. As a result, when mitochondria were impaired, dysfunction occurred [22]. However, physiological net effect on mitochondrial function is still unclear.

Mitochondrial biogenesis is regulated by a network of signaling factors. The peroxisome proliferated receptor gamma co-activator (PCG) family of transcription co-activators (PCG-1α/β) is a major group of regulators involved in mitochondrial biogenesis an important regulator of oxidative metabolism in the heart [23]. PCG-1α binds to and co-activates several transcriptional factors at their nuclear encoded gene promoter regions. PCG-1α co-activates nuclear respiratory factor-1 (NRF) and NRF-2 to upregulate the expression of almost all nuclear encoded mitochondrial genes, which are involved in oxidative phosphorylation (OXPHOS). Furthermore, PCG-1α indirectly induces mitochondrial DNA replication and transcription by increasing expression of mitochondrial DNA regulatory genes, including mitochondrial transcription factor A (TFAM), Tu translation elongation factor mitochondria (TUFM), single strand binding protein (SSBP), transcription factor B2 (TFB2M), and mitochondrial DNA direct RNA polymerase (POLRMT) [24,25,26]. Consequently, mitochondrial biogenesis function increases when PCG-1α is activated. Numerous studies have reported that natural phytochemicals, such as the well-known resveratrol, regulate mitochondrial biogenesis [27,28,29,30,31].

In this study, we focus on the natural compound Ech A, which has a beneficial effect on cardiac mitochondrial biogenesis and OXPHOS function without cytotoxicity. We investigated how Ech A affects cardiac mitochondrial function in cardiomyocytes via the PGC-1α signal pathway by measuring changes in downstream gene expression after treatment with Ech A.

## 2. Results and Discussion

### 2.1. Echinochrome A Reduced ROS Generation, but Did Not Interfere with Cellular Viability

**Figure 1:**
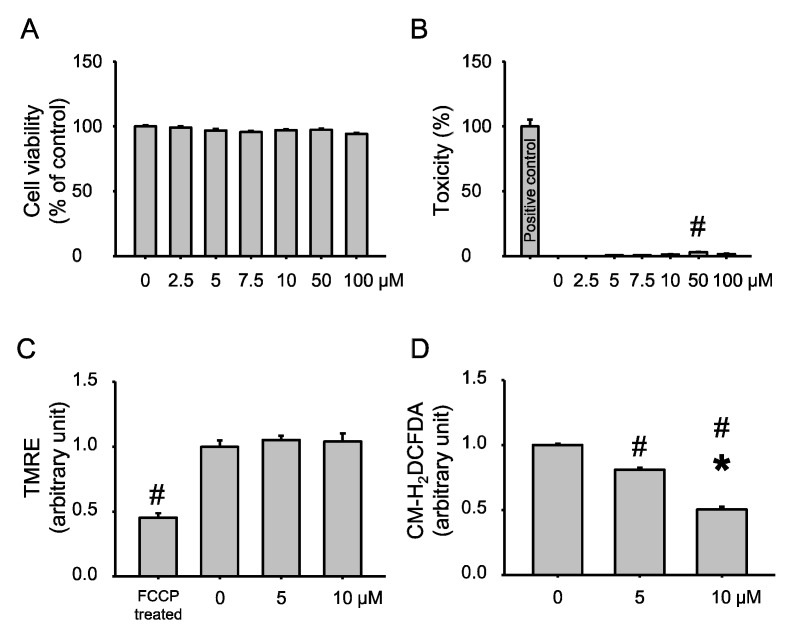
Echinochrome A reduced reactive oxygen species (ROS) generation but did not interfere with cellular viability. (**A**) Rat cardio myoblast H9c2 cells were treated with Ech for 24 h and then tested for cell viability with an MTT assay; (**B**) Toxicity was measured using Cell Tox™ Green. For positive control we applied provided lysis solution by manufacturer. The lysis solution induced cell permeability, resulting in maximal cell death; (**C**) Mitochondrial membrane potential (ΔΨm; TMRE); and (**D**) Reactive oxygen species (ROS; CM-H_2_DCFDA) were measured by flow cytometer. # *p* < 0.05 non-treated *vs.* treated. * *p* < 0.05 between treated group.

First, we investigated whether Ech A influences cell proliferation and cell viability using rat cardio myoblast H9c2 cells. The cells were cultured with 0, 2.5, 5, 7.5, 10, 50, and 100 μM of Ech A for 24 h. As Figure 1 shows, Ech A did not influence the cell viability (Figure 1A) and doses lower than 50 μM did not display toxicity in H9c2 cells (Figure 1B). We selected 2 doses of Ech A (5 and 10 μM) and applied in the next experiment. Ech A did not alter mitochondrial membrane potential (Figure 1C). However, Ech A reduced cellular reactive oxygen species (ROS) significantly (Figure 1D). Previous studies on Ech A described that Ech A is not only powerful superoxide anion-radical scavenger [32], but also has a cardioprotective activity from cardiotoxic drugs [4,33,34]. These results suggest that Ech A does not interfere with the cell viability in rat cardio myoblast H9c2 cells.

### 2.2. Echinochrome A Enhanced Mitochondrial Biogenesis Function

**Figure 2:**
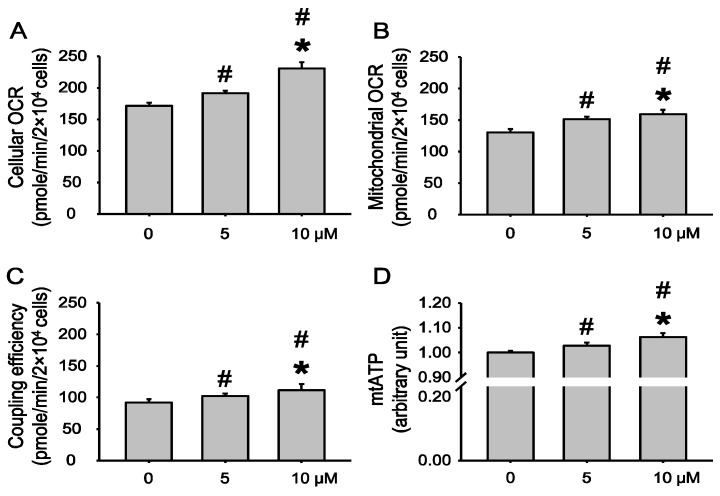
Echinochrome A enhances mitochondrial biogenesis function. Rat cardio myoblast H9c2 cells were treated with Ech A for 24 h. Cellular Oxygen Consumption Rate (OCR) (**A**); mitochondrial OCR (**B**); and coupling efficiency (**C**) were measured using a XF24 analyzer. To confirm OCR data, we measured mitochondrial ATP level (**D**). # *p* < 0.05 non-treated *vs.* treated. * *p* < 0.05 between treated groups.

Next, we investigated whether Ech A influences oxidative phosphorylation and energy production. We measured cellular oxygen consumption rate (OCR) 24 h after Ech A treatment. Interestingly, Ech A treatment significantly increased cellular oxygen consumption rate (Figure 2A). Cells consume the most oxygen at the glycolysis and oxidative phosphorylation stages. Therefore, we tested whether the increased cellular OCR could be attributed to the oxidative phosphorylation stage. To determine mitochondrial OCR and coupling efficiency, we used a XF24 analyzer and its application Mitostress, which analyzes mitochondrial OCR and coupling efficiency from the cell. As shown in Figure 2B, mitochondrial OCR was significantly increased by Ech A in a dose-dependent manner. Likewise, the coupling efficiency and ATP synthetic efficiency were increased after Ech A treatment in a dose-dependent manner (Figure 2C). To confirm coupling efficiency, we measured mitochondrial ATP level. As shown in Figure 2D, mitochondrial ATP level was significantly increased by Ech A treatment in a dose-dependent manner.

**Figure 3:**
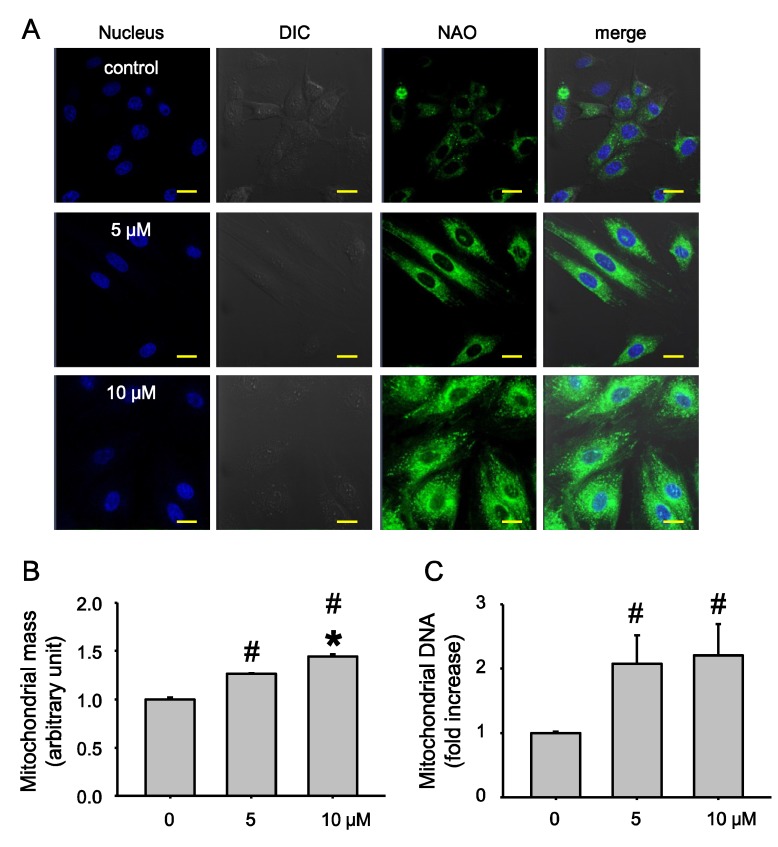
Echinochrome A increases mitochondrial contents. Rat cardio myoblast H9c2 cells were treated with Ech A for 24 h, and then mitochondrial mass and contents were analyzed using confocal microscopy and flow cytometry. (**A**) Mitochondrial mass (10-nonylacridine orange (NAO) intensity) was increased in Ech A treated cells; (**B**) Enhanced NAO intensity cells were increased by Ech A treatment; (**C**) Mitochondrial DNA contents were increased significantly by Ech A treatment. # *p* < 0.05 non-treated *vs.* treated. * *p* < 0.05 between treated groups. Scale bar = 20 μm.

Next, we investigated whether the observed increase in mitochondrial function was due to increased mitochondrial contents. We measured mitochondrial contents using 10-nonylacridine orange (NAO) staining and confocal microscopy. We found that mitochondrial mass as measured by NAO intensity, was increased by Ech A treatment (Figure 3A, quantified in Figure 3B). This result was confirmed by flowmeter analysis. Similar to the results of confocal image analysis, Ech A treatment increased mitochondrial mass in a dose-dependent manner. To determine whether Ech A treatment increased mitochondrial DNA content, we measured mitochondrial DNA content (Figure 3C). Mitochondrial DNA contents were also increased by Ech A treatment. These data suggest that Ech A treatment effectively elevated mitochondrial biogenesis and oxidative phosphorylation.

### 2.3. Echinochrome A Upregulated Mitochondrial Biogenesis Related Genes

**Figure 4:**
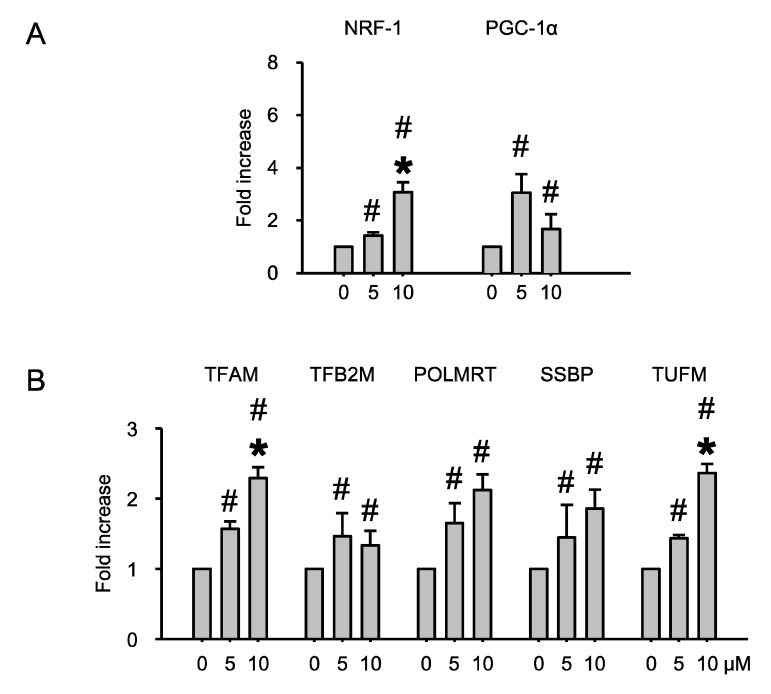
Echinochrome A upregulated expression of mitochondrial biogenesis regulated gene. (**A**) Ech A treatment increased expression of mitochondrial biogenesis-regulated transcriptional factor; (**B**) Mitochondrial DNA transcriptional factors were also increased by Ech A treatment. # *p* < 0.05 non-treated *vs.* treated. * *p* < 0.05 between treated group.

To further evaluate mitochondrial biogenesis, we examined mitochondrial biogenesis-regulated gene expression levels by quantitative real-time PCR. As describe in previous studies, mitochondrial biogenesis is regulated by various transcription factors, such as PGC-1α and NRF-1 [35,36,37,38]. These factors increase OXPHOS regulation proteins and enhance mitochondrial DNA transcription, either directly or indirectly. As shown in Figure 4A, these genes were increased by Ech A in a dose-dependent manner. Likewise, mitochondrial DNA transcriptional regulation factor genes, including TFAM, TFB2M, POLMRT, SSBP, and TUFM, were also activated (Figure 4B). TFAM, TFB2M and SSBP increased mitochondrial DNA or RNA transcription [39,40] and POLMRT and TUFM increased OXPHOS component proteins [41]. The dose-dependent stimulation of NRF-1 expression appears to be an important property of Ech A action on myoblast H9c2 cells. In fact, NRF-1 is a DNA-binding regulator of mtDNA transcription. Moreover, it regulates many other aspects of mitochondrial function. NRF-1 binding sites are present in promoters of cytochrome c and other mitochondrial genes that are encoded at different stages of oxidative phosphorylation.

It seems plausible that the effects of Ech A, such as increasing of oxygen consumption rate (Figure 2), are consequences of NRF-1 upregulation. NRF-1 activity should increase because of interaction with the PGC-1α co-factor. Interestingly, we observed upregulation of PGC-1α upon Ech A treatment. Remarkably, this upregulation was more significant at the low dose of Ech A. On the other hand, the increase of OCR after Ech A treatment (Figure 2) may be connected to its upregulatory effect on genes that encode other mtDNA transcription proteins, such as TFAM and TFB2M. Dose-dependent upregulation of mitochondrial POLMRT shows that the action of Ech A covers both mtDNA transcription and replication, as evidenced by the increased mtDNA mass as well as in the increase in the number and size of mitochondria (Figure 3). It is well known that incompetence of the polymerase, as a consequence of mutations of the corresponding gene, is associated with several mitochondrial diseases such as progressive ophtalmoplegia, Alpers’ disease, and others. Ech A may be useful as a biopreparation, which attenuates symptoms of mitochondrial diseases of some patients. The observed upregulation of SSBP suggests a positive influence of Ech A treatment on replication processes as well. Finally, dose-dependent up-regulation of TUFM by Ech A suggests that this drug may stimulate some stages of translation, which partly explains some of the morphological changes observed in the mitochondria (Figure 3). Thus, Ech A functions not only as classical antioxidant, but also alters dramatically the biochemical process of mitochondria, stimulating mitochondrial energy metabolism.

**Figure 5:**
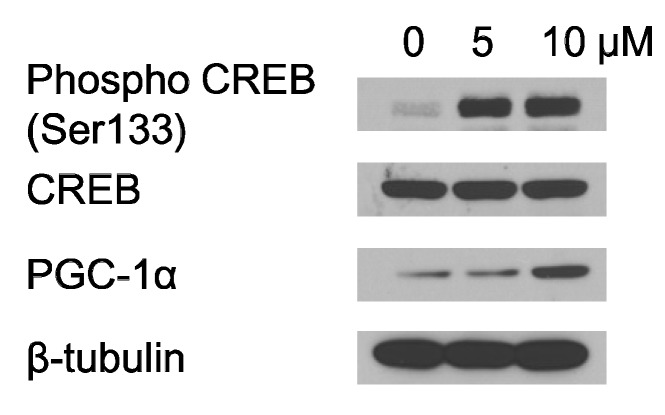
Echinochrome A modulated proliferator-activated receptor gamma co-activator (PGC)-1α expression via phosphorylation of CREB. Ech A treatment increased phosphorylation of CREB. Also PGC-1α was increased.

One of the important activators, CREB (cAMP response element-binding protein), increases expression of PGC-1α [25,42]. Therefore, western blot analysis was performed to determine whether PGC-1α is activated through CREB during Ech A treatment. As shown in Figure 5, Ech A treatment increased phosphorylation of CREB and expression of PCG-1α. These results suggest that Ech A treatment enhances mitochondrial DNA transcriptional regulation genes through upregulation of mitochondrial biogenesis transcription genes.

## 3. Experimental Section

### 3.1. Chemicals

Ech A was isolated from the sea urchin *Scaphechinus mirabilis* (Agassiz) by a previously described method [43]. The purity of Ech A (99.0%) was confirmed by LS-MS data (Shimadzu LCMS-2020, Kyoto, Japan). Purified Ech A appeared like red-brown needles, was soluble in Ethanol, had a melting point of 219 °C–221.5 °C (lit. 220 °C–221 °C [44]); and similar NMR spectra to that reported previously [43]. We used a solution of 750 μM Ech A di-sodium salts (Histochrome^®^), produced by Pacific Institute of Bioorganic Chemistry, Far East Branch of the Russian Academy of Sciences as a stock solution [11].

### 3.2. Cell Culture

Rat cardio myoblast H9c2 cells (American Type of Culture collection, Manassas, VA, USA) were maintained in Dulbecco’s Modified Eagle Medium supplemented with 10% of heat-inactivated fetal bovine serum, 50 U/mL penicillin and 50 μg/mL streptomycin (all from Lonza, Walkersville, MD, USA).

### 3.3. Measurement Cell Viability

H9c2 cells were plated 2 × 10^4^ cells/well in 96-wells tissue culture plate. After 16 h, the cells were treated with 0, 5, or 10 μM of Ech A for 24 h. Cell viability was measured by quantitative colorimetric assay with MTT (3-(4,5-dimethylthiazol-2-yl)-2,5-diphenyltetrazolium bromide; Sigma-Aldrich, St. Louis, MO, USA), which measures the mitochondrial activity of living cells. The extent of reduction of MTT to formazan within the cells was quantified by measuring the optical density at 570 nm using a microplate reader (Molecular device, Sunnyvale, CA, USA).

### 3.4. Measurement of Cytotoxicity

H9c2 cells were plated 2 × 10^4^ cells/well in black and clear bottom 96-wells tissue culture plate. After 16 h, the cells were treated with 0, 2.5, 5, 7.5, 10, 50, and 100 μM of Ech A for 24 h. Cytotoxicity was assessed by quantitative fluorescence assay with CellTox Green cytotoxicity assay (Promega, Madison, WI, USA). This cytotoxicity assay measures changes in membrane integrity that occur as results of cell death. For a positive control (as maximal cell death), lysis buffer (as 0.02% digitonin) was added in non-treated cells. Cells were quantified by measuring fluorescence (excitation/emission = 485 nm/530 nm) using a microplate reader (Molecular Device, Sunnyvale, CA, USA).

### 3.5. Measurement of Mitochondrial Membrane Potential

To evaluate the effect of Ech A in mitochondria, mitochondrial inner membrane potential (ΔΨm) was compared in cells treated with 0, 5, or 10 μM of Ech A using the fluorescent dye tetramethylrhodamine, ethyl ester (TMRE; excitation/emission = 549 nm/574 nm; Invitrogen, Carlsbad, CA, USA) which is sequestered by active mitochondria. H9c2 cells were plated 2 × 10^6^ cells in 60 mm cell culture plate. After 16 h, the cells were treated with 0, 5, or 10 μM of Ech A for 24 h. Then the cells were stained with 200 nM TMRE for 30 min at 37 °C. Cells were washed twice with PBS, and then the relative signal intensity of TMRE in cells was analyzed using a FACsCalibur flow cytometer (BD Biosciences, San Jose, CA, USA).

### 3.6. Measurement of ROS

To evaluate the effect of Ech A in mitochondria, ROS was measured in cells treated with Ech A (0, 5 or 10 μM) using the fluorescent dye CM-H_2_DCF-DA (excitation/emission = 492_nm_/517_nm_; Invitrogen, Carlsbad, CA, USA), a general ROS indicator. H9c2 cells were plated 2 × 10^6^ cells in 60 mm cell culture plate. After 16 h, the cells were treated with 0, 5, or 10 μM of Ech A for 24 h. The cells were stained with 10 μM CM-H_2_DCF-DA for 30 min at 37 °C. After washing twice with PBS, relative signal intensity of CM-H_2_DCF-DA in cells was analyzed using a FACsCalibur flow cytometer (BD Biosciences, San Jose, CA, USA).

### 3.7. Measurement of Mitochondrial ATP Level

Mitochondrial ATP level was measured by Mitochondrial ToxGlo™ assay (Promega, Madison, WI, USA) according to the manufacture’s protocol. Briefly, H9c2 cells were plated 2 × 10^6^ cells/well in 60 mm tissue culture plate. After 16 h, the cells were treated with 0, 5, or 10 μM of Ech A for 24 h. Treated cells were harvested and resuspended by pipetting until the cells were evenly dispersed. Resuspended H9c2 cells were plated 2 × 10^4^ cells/well in white and clear bottom 96-well culture plate. Plates were centrifuged at 200× g for 10 min to remove medium and after it 50 μL of fresh medium lacking glucose and supplemented with 10 mM galactose. The plate incubated at 37 °C in a humidified and CO_2_-supplemented incubator for 90 min. Assay solution (100 μL) was added to the plate, and then the plate was incubated at room temperature for 30 min. Luminescence was measured using a luminometer (Molecular Device, Sunnyvale, CA, USA).

### 3.8. Measurement of Oxygen Consumption Rate (OCR)

OCR was measured using a XF24 analyzer (Seahorse Bioscience, Billerica, MA, USA) as previously described [38]. Briefly, H9c2 cells were plated 2 × 10^4^ cells/well in XF24 cell culture plate (Seahorse Bioscience, Billerica, MA, USA). After 16 h, the cells were treated with various doses of Ech A. After 24 h, the medium was exchanged for 500 μL of XF Assay Medium-modified DMEM (Seahorse Bioscience, Billerica, MA, USA) and then incubated at 37 °C without CO_2_ for 1 h. OCR was measured by XF24 analyzer and XF24 software. After measuring the OCR, the XF24 assay results were normalized to cell number. Cell number for each well was counted using a Luna™ automated cell counter (Logos, Annandale, VA, USA).

### 3.9. Measurement of Mitochondrial Mass

To measure the mitochondrial mass, cells treated with 0, 5, or 10 μM of Ech A were stained with acridine orange 10-nonyl bromide at a final concentration of 2.5 μM in phosphate buffered saline (PBS) (NAO; Invitrogen, Carlsbad, CA, USA) This dye binds with mitochondrial membrane cardiolipin. Importantly, binding is independent of ΔΨm over the physiologically relevant range. The cells were incubated in dark at 37 °C for 30 min then washed twice with PBS. NAO fluorescence for the each group was measured in laser confocal microscope (LSM 700, Carl-Zeiss, Oberkochen, Germany). The cells were excited using 488 nm and emission of NAO was measured beyond 585 nm. The mean intensity of the region of interest (ROI) was measured in each cell and analyzed by ZEN2009 software (Carl-Zeiss, Oberkochen, Germany). Nucleoli of cells were co-stained with Hoechst 33342 as a counter stain.

To confirm the mitochondrial mass, H9c2 cells were plated 2 × 10^6^ cells in 60 mm cell culture plate. After 16 h, the cells were treated with 0, 5, or 10 μM of Ech A for 24 h. The cells were stained with 2.5 μM NAO for 30 min at 37 °C. After washing twice with PBS, relative signal intensity of NAO in cells was analyzed using a FACsCalibur flow cytometer (BD Biosciences, San Jose, CA, USA).

### 3.10. Reverse Transcription Polymerase Chain Reaction (RT-PCR) and Real-Time PCR

Total RNA of H9c2 cells was extracted using Trizol reagent (Invitrogen, Carlsbad, CA, USA). One microgram of total RNA was reverse transcribed using Revet Aid First Strand cDNA Synthesis kit (Thermo, Rockford, IL, USA) according to the manufacturer’s instructions. The forward and reverse primers are shown in Table 1. Real-time PCR was carried out using SYBR premix Ex Taq (Takara, Shiga, Japan). Reactions were prepared following the manufacturer’s protocol. All reactions were carried out in triplicate (Bio-Rad, Hercules, CA, USA). The cDNA was amplified through 60 cycles of 15 s at 95 °C, 30 s at 58 °C and 30 s at 72 °C for each gene. Data analysis was carried out using CFX manager™ software (Bio-Rad, Hercules, CA, USA) and Microsoft Excel. Expression values are presented relative to the measurements for beta-tubulin values in the corresponding samples.

**Table 1:** Gene primers used in this study.

Gene	Forward Primer	Reverse Primer
NFR-1	ATTATTCTGCTGTGGCTGATG	CGTCGTCTGGATGGTCAT
PGC-1α	CACCGTATTTGAGGACAGCA	GAAGTTCTTCCGGGTAGCTG
TFAM	AGAGTTGTCATTGGGATTGG	CATTCAGTGGGCAGAAGTC
TFB2M	GCATTGATTTGGGCAGAC	AACTGGCATTGAACTGGT
PLMRT	AGAGTGCCAACCTCATCTCT	CAGGGAGTGGATGAAGTTGG
SSBP	GGGCTCGTATATTTGTGGAA	GCTATGATTGTTGTTGCTTGC
TUFM	CCCTTTCTGCTCCCTGTA	CAACTCACACTCATCTCCTT
β-Tubulin	GTTTTGGGAGGTCATCAGTG	CCAGTTATTTCCTGCACCAC
d-Loop(mtDNA)	ATCCTCCGTGAAATCAACAA	CAGGACTTTGTGCTGACCTT
B2M(chDNA)	CCCAACTTCCTCAACTGCTA	GCTCCTTCAGAGATGACGTGT

### 3.11. Quantitative PCR for Mitochondrial DNA

Total DNA, including chromosomal and mitochondrial DNA, was extracted from H9c2 cells treated with 0, 5, or 10 μM Ech A using a Gentra Puregene kit (Qiagen, Hilden, Germany) following the manufacturer’s instructions. The forward and reverse primers are shown in Table 1. Real-time PCR was carried out SYBR Premix Ex Taq (Takara, Shiga, Japan). Total DNA was amplified through 60 cycles of 15 s at 95 °C, 30 s at 58 °C, and 30 s at 72 °C for each gene. Data analysis was carried out using CFX manager™ software (Bio-Rad, Hercules, CA, USA) and Microsoft Excel. Expression values are presented relative to the measurements for beta-tubulin values in the corresponding samples.

### 3.12. Western Blot Analysis

Cell lysates were centrifuged at 14,000 rpm for 15 min at 4 °C. Protein concentrations were determined by Bradford protein assay (Bio-Rad, Hercules, CA, USA), and 30 μg of protein was loaded per lane onto 10% SDS polyacrylamide gels. Gels were transferred onto nitrocellulose membranes (Whatman, Freiburg, Germany) and incubated with specific antibodies (CREB, pCREB (Ser133), and beta-tubulin; Cell Signaling, Danvers, MA, USA, PGC-1; Santa Cruz Biotechnology, Santa Cruz, CA, USA). Western blot analysis was performed using these antibodies and a western blotting detection kit, Ab signal™ (AbClon, Seoul, Korea). Blots were visualized with an LAS-3000 Plus imager (Fuji Photo Film Company, Tokyo, Japan).

### 3.13. Data Analysis

Unless stated otherwise, all experiments were performed in triplicate. Data are presented as means ± standard error of the mean (SEM). One-way ANOVA was used to compare values between groups, and *p* ≤ 0.05 was considered significant.

## 4. Conclusions

To the best of our knowledge, this is the first study that investigates the net effect of Ech A on mitochondrial biogenesis and OXPHOS function. Our results indicate that Ech A increased mitochondrial mass and OXPHOS function significantly, which enhanced mitochondrial energy efficiency by modulating major mitochondria biogenesis regulatory genes, including PGC-1α and NRF-1. These results provide some evidence that Ech A has the potential to enhance mitochondrial energy metabolism, which may be clinically beneficial for the treatment of various mitochondrial dysfunctions implicated in metabolic diseases. These results can explain the ATP-saving effect of the Histochrome^®^ drug used to treat acute myocardial ischemia in patients [39]. In future studies based on these results, we plan to test this hypothesis using pre-clinical models, including *ex vivo* and animal models.
